# Food Insecurity and Cardiovascular-Kidney-Metabolic Syndrome–Related Conditions Among US Adults, National Health and Nutrition Examination Survey, 2015–2020

**DOI:** 10.5888/pcd23.250372

**Published:** 2026-08-27

**Authors:** Deepali K. Ernest, Bipin Singh, Aydee Hinojosa, Alveena Imran, Elizabeth A. Onugha, Shreela V. Sharma, Jayna M. Dave

**Affiliations:** 1 Department of Epidemiology, University of Texas Health Science Center at Houston, School of Public Health, Houston, Texas; 2 Center for Health Equity, University of Texas Health Science Center at Houston, School of Public Health, Houston, Texas; 3 Department of Epidemiology, Peter O’Donnell Jr. School of Public Health, UT Southwestern Medical Center, Dallas, Texas; 4 Baylor College of Medicine, Houston, Texas; 5 University of Houston, Houston, Texas; 6 Department of Pediatrics, Texas Children’s Hospital, Houston, Texas; 7 Department of Pediatrics, Baylor College of Medicine, Houston, Texas; 8 Texas Children’s Research Institute, Houston, Texas; 9 US Department of Agriculture, Agricultural Research Service, Children’s Nutrition Research Center, Houston, Texas

## Abstract

**Introduction:**

Cardiovascular-kidney-metabolic (CKM) syndrome includes health disorders that are increasingly prevalent in the US, yet their relationship with food insecurity remains unclear. This study examined CKM syndrome–related conditions among US adults and their associations with food insecurity.

**Methods:**

This cross-sectional study analyzed data for 13,937 US adult participants from the 2015–2020 National Health and Nutrition Examination Survey. Information about demographic characteristics, food insecurity, and CKM syndrome–related conditions was self-reported on questionnaires. Weighted logistic regression models, adjusting for demographic variables and socioeconomic status, examined associations between food insecurity and CKM syndrome–related health outcomes.

**Results:**

Mean age of respondents was 47.4 years, and mean body mass index (weight in kg/height in m^2^) was 29.6. Of total respondents, 28.4% experienced marginal, low, and very low food security. Obesity (40.9%), hyperglycemia (58.9%), and hypertension (29.9%) were the most prevalent cardiometabolic conditions. Lower food security was associated with significantly higher odds of obesity, diabetes, weak or failing kidneys, and cardiovascular diseases (CVDs), with clear dose–response relationships. Compared with full food security, very low food security was associated with higher odds of congestive heart failure (adjusted odds ratio [AOR] = 3.69), coronary heart disease (AOR = 3.43), heart attack (AOR = 3.21), and stroke (AOR = 2.54). Additionally, the likelihood of low or very low food security increased stepwise with number of coexisting CVDs, with the highest odds observed among adults reporting 4 CVDs (AOR = 3.93).

**Conclusion:**

This study highlights that food insecurity is significantly associated with CKM syndrome–related conditions among US adults. Addressing food insecurity is critical to reducing its substantial health burden and improving population health.

**Summary**

**What is already known on this topic?**

Food insecurity is a key social determinant of health that is linked to diabetes, hypertension, and cardiovascular disease. However, its impact on cardiovascular-kidney-metabolic (CKM) syndrome–related conditions is not fully known.

**What is added by this report?**

Our study results demonstrated markedly higher odds of obesity, diabetes, weak or failing kidneys, and various cardiovascular events among food-insecure versus food-secure populations.

**What are the implications for public health practice?**

Findings from our study provide a strong foundation for larger and more robust longitudinal studies to further examine food insecurity as a risk factor for CKM syndrome–related conditions.

## Introduction

Chronic cardiovascular, metabolic, and kidney diseases are among the leading causes of death globally (1). Cardiovascular-kidney-metabolic (CKM) syndrome is a newly established disorder defined as the clinical manifestation of interactions between metabolic risk factors (eg, obesity, diabetes), chronic kidney disease (CKD), and the cardiovascular system (2). Currently, the progression of CKM syndrome is measured from stage 0 (absence of risk factors) to stage 4 (severe organ damage, such as heart disease or weak or failing kidneys) (3). Its development is influenced not only by biologic processes but also by key systemic factors, including socioeconomic inequalities, environmental barriers, and policy-related challenges that limit the adoption of healthy lifestyle practices (2). CKM syndrome–related conditions have severe clinical implications, including increased risk of cardiovascular events and associated death (3). Despite growing recognition, gaps remain in the understanding of its risk factors, particularly social risk factors such as food insecurity and socioeconomic disadvantage.

Food insecurity, defined as inconsistent access to adequate food for an active and healthy lifestyle, is a key social determinant of health (4). Prior studies have linked prolonged food insecurity to the onset of common cardiometabolic disorders such as type 2 diabetes and hypertension (4,5). Evidence also demonstrates that food-insecure people have nearly twice the odds of developing cardiovascular disease (CVD) compared with food-secure people (5). Te Vazquez et al described 3 primary pathways —nutritional, compensatory, and psychologic — through which food insecurity is associated with adverse health outcomes (6). Although the role of food insecurity in cardiometabolic disorders among US adults has been studied, little is known about its contribution to CKM syndrome–related conditions. Given the rising prevalence and consequences of CKM syndrome–related conditions, understanding if and how food insecurity is linked to their development is critical to designing public health interventions. Therefore, this study aimed to address this gap in the literature by cross-sectionally examining the relationship between food insecurity and CKM syndrome–related conditions among a diverse and nationally representative sample of US adults.

## Methods

### Study data and population

This cross-sectional analysis used data for 13,937 adults enrolled in the National Health and Nutrition Examination Survey (NHANES) during the 2015–2016 cycle and the cycle from 2017 through March 2020. Participants who were aged 18 years or younger (n = 9,846) or had missing food insecurity data (n = 1,748) were excluded from the original NHANES sample (N = 25,531), resulting in a final sample of 13,937 adults. All participants provided informed consent upon enrollment into NHANES. The University of Texas Health Science Center Internal Review Board deemed this study to be exempt from review due to the use of publicly available, deidentified data.

### Assessment of CKM syndrome–related conditions

CKM syndrome–related conditions were identified by using the adult definition established by the American Heart Association (7) and included obesity (body mass index [BMI, weight in kg/height in m^2^] ≥30), type 2 diabetes, hyperglycemia, weak or failing kidneys, hypertension, and CVDs (ie, congestive heart failure, coronary heart disease, angina, heart attack, and stroke). Participants’ body measurements and bio-specimens were collected by trained NHANES personnel (8). For this study, NHANES-generated BMI values were used to generate weight categories based on cutoffs established by the Centers for Disease Control and Prevention (9). Participants who had a BMI of 30 or higher were considered to have obesity. Additionally, participants’ blood pressure was measured 3 consecutive times to account for within-person variations in measurements (10). If any measurements were incomplete or inconclusive, a fourth reading was taken to ensure the completion and accuracy of blood pressure data. For this study, the average systolic blood pressure and diastolic blood pressure readings were calculated for all participants. Participants who had an average systolic blood pressure of 130 mm Hg or higher or an average diastolic blood pressure of 80 mm Hg or higher were considered to have hypertension (11). Lastly, hyperglycemia was defined as having a plasma glucose concentration of 100 mg/dL or higher, which included participants in the prediabetes range (≥120 mg/dL) (12). Enzymatic and photometric methods were used to measure serum fasting glucose levels from participants’ blood samples, which is further detailed in the NHANES laboratory procedure manual (13). Each metabolic outcome was treated as a dichotomous variable (1 = condition present, 0 = condition not present).

Other cardiovascular clinical events were self-reported on NHANES health questionnaires. Participants were asked to report whether they had “ever been told by a doctor or health professional that they had diabetes or sugar diabetes”; their responses were recorded as either yes, no, or “borderline” (14). For this study, participants who responded yes or “borderline” were considered to have diabetes (diabetes = 1) while others were not (diabetes = 0). Similarly, participants were asked to report whether they had “ever been told by a doctor or other health professional that they had weak or failing kidneys,” not including kidney stones, bladder infections, or incontinence (15). Participants’ responses were recorded as either yes or no by NHANES and retained as a dichotomous variable for this study. On the Medical Conditions Questionnaire, participants were asked to report whether they were told by a doctor or health care professional that they had certain CVDs (ie, congestive heart failure, coronary heart disease, angina, heart attack, and stroke) (16); their responses were recorded as either yes or no and retained as individual, dichotomous variables for this study. Participants who refused to provide responses to these questions or were unable to recall or provide the relevant information were treated as having missing data; missing observations were considered missing at random and excluded from analysis.

### Assessment of food security

Food security status was assessed by NHANES by using the 18-item US Food Security Survey Module for households with children younger than 18 years and by using a shortened 10-item version for households without children (17,18). Participant responses were scored by NHANES staff and tallied to generate a raw food security score. A 4-level household food security variable was created, with 1 being full food security, 2 being marginal food security, 3 being low food security, and 4 being very low food security, which was retained for our analysis. 

### Assessment of participant demographics

Participants’ age, sex, race and ethnicity, and socioeconomic status (SES) were self-reported on the NHANES demographics questionnaire or collected during in-person interviews (8). Family poverty index, a proxy measure of SES, was calculated by dividing total annual family income by the poverty guidelines set by the US Department of Health and Human Services specific to the survey year (19). Participants with a family poverty index of 1.85 or less were classified as having low SES, consistent with cutoffs used to determine financial eligibility for many federal programs (19).

### Statistical analysis

Participant characteristics were reported as mean (SE) for continuous variables and frequency (weighted %) for categorical variables. Stratified analyses and cross-tabulations were used to determine the weighted prevalence of each symptom of CKM syndrome across food security levels. A composite CVD outcome variable was created, summing participant responses (0 = condition not present, 1 = condition present) for each CVD. This score was created to reflect the total number of CVDs reported by each participant across the entire study sample. The adjusted associations between food security state, CKM symptom–related conditions, and cumulative CVD score were examined by using weighted logistic regression models, accounting for the complex sampling design of NHANES. Multivariable models were adjusted for participants’ age, sex, race and ethnicity, and SES. These covariates were chosen a priori, based on their established relationship with food insecurity (20,21). The type 1 error level was set at 5%, and a *P* value < .05 was considered significant. All analyses were performed by using SAS Studio version 9.4 (SAS Institute, Inc).

## Results

Participants had a mean age of 47.4 years (SE = 0.4 y) and a mean BMI of 29.6 (SE = 0.2). Of all participants, 51.9% were female, 15.8% Hispanic, 63.3% non-Hispanic White, 11.3% non-Hispanic Black, 5.7% non-Hispanic Asian, and 3.9% other or multiple races (Table 1). Nearly 14% came from households at or below the poverty line, 11.2% had marginal food security, 9.5% low food security, and 7.7% very low food security. The most prevalent cardiometabolic condition was hyperglycemia (58.9%), followed by obesity (40.9%) and hypertension (29.9%). Less-common conditions included type 2 diabetes (13.5%) and weak or failing kidneys (3.2%) (Table 1). The distribution of participants across NHANES survey years is presented in the Appendix.

**Table 1:** Distribution of Participant Demographics and CKM Syndrome–Related Conditions, by Food Security Status (N = 13,937), NHANES, 2015–2020^a^

Characteristic	Overall (N = 13,937)	Food security status^b^				*P* value^c^
		Full (n = 8,604)	Marginal (n = 2,051)	Low (n = 1,862)	Very low (n = 1,420)	
Age, mean (SE), y	47.4 (0.4)	49.1 (0.5)	43.2 (0.7)	43.8 (0.8)	42.3 (0.8)	<.001
BMI, mean (SE), kg/m^2^	29.6 (0.2)	29.2 (0.2)	30.3 (0.3)	30.6 (0.3)	30.8 (0.4)	<.001
**Sex**						
Male	6,726 (48.1)	4,227 (48.9)	954 (46.0)	860 (45.2)	685 (46.9)	.01
Female	7,211 (51.9)	4,377 (51.1)	1,097 (54.0)	1,002 (54.8)	735 (53.1)	
**Race and ethnicity**						
Hispanic	3,548 (15.8)	1,709 (10.9)	653 (26.8)	744 (33.9)	442 (23.3)	<.001
Non-Hispanic Asian	1,634 (5.7)	1,271 (6.3)	190 (5.6)	147 (4.8)	26 (0.9)	
Non-Hispanic Black	3,400 (11.3)	1,832 (8.8)	649 (18.9)	493 (16.8)	426 (17.2)	
Non-Hispanic White	4,734 (63.3)	3,459 (70.7)	456 (42.9)	385 (39.7)	434 (52.4)	
Other/multiple races	621 (3.9)	333 (3.3)	103 (5.7)	93 (4.7)	92 (6.2)	
**Family income-to-poverty ratio**						
At or below poverty line	2,758 (13.8)	892 (6.3)	550 (24.3)	666 (35.0)	650 (43.9)	<.001
Above poverty line	10,080 (86.2)	7,096 (93.7)	1,297 (75.7)	995 (65.0)	692 (56.1)	
**Cardiometabolic disorders**						
Obesity	5,674 (40.9)	3,260 (38.6)	893 (45.3)	856 (47.3)	665 (47.6)	<.001
Type 2 diabetes	2,391 (13.5)	1,356 (12.7)	370 (15.0)	385 (16.1)	280 (14.9)	.02
Hyperglycemia	3,881 (58.9)	2,326 (58.9)	602 (61.5)	573 (60.4)	380 (57.9)	.63
Weak or failing kidneys	546 (3.2)	288 (2.8)	105 (4.1)	87 (4.1)	66 (5.0)	.001
Hypertension	4,428 (29.9)	2,748 (29.8)	642 (31.0)	578 (29.6)	460 (29.6)	.91
Congestive heart failure	498 (2.6)	266 (2.2)	78 (2.6)	78 (3.4)	76 (4.6)	<.001
Coronary heart disease	586 (3.9)	362 (3.9)	74 (3.5)	73 (3.4)	77 (5.7)	.10
Angina	335 (2.3)	181 (2.1)	49 (2.2)	60 (3.3)	45 (2.7)	.06
Heart attack	613 (3.6)	340 (3.2)	85 (3.7)	91 (4.4)	97 (5.9)	<.002
Stroke	612(3.4)	332 (3.0)	87 (2.8)	107 (4.8)	86 (5.7)	<.001

Abbreviations: BMI, body mass index; CKM syndrome, cardiovascular-kidney-metabolic syndrome; NHANES, National Health and Nutrition Examination Survey.

a Values are no. (weighted %), unless otherwise indicated.

b Food security status was assessed by using the 18-item US Food Security Survey Module for households with children younger than 18 years and a shortened 10-item version for households without children. Participant responses were scored by NHANES staff and tallied to generate a raw food security score. A 4-level household food security variable was created, with 1 being full food security, 2 being marginal food security, 3 being low food security, and 4 being very low food security.

c Significance set at *P* < .05.

Compared with participants with full food security, those with very low food security were, on average, younger and had a higher BMI (Table 1). Additionally, a larger proportion of this group also came from households at or below the poverty line; were obese; had weak or failing kidneys; and had congestive heart failure, coronary heart disease, heart attack, or stroke compared with those with full, marginal, or low food security. Most participant demographics and cardiometabolic disorders were significantly associated with food security except for hyperglycemia (*P* = .63), hypertension (*P* = .91), coronary heart disease (*P* = .10), and angina (*P* = .06) (Table 1).

After adjusting for age, sex, race and ethnicity, and SES, lower levels of food security were significantly associated with most metabolic disorders, with significant dose–response relationships observed, especially for obesity and diabetes (Table 2). Compared with participants with full food security, the likelihood of having obesity and diabetes was significantly greater among those with very low food security (42% and 64%, respectively) than among those with marginal food security (33% and 60%, respectively). The AOR for having weak or failing kidneys was 1.70 for marginally food-secure participants and 2.26 among participants with very low food security, compared with those with full food security.

**Table 2:** Adjusted Associations Between Food Insecurity and Symptoms of CKM Syndrome–Related Conditions, NHANES, 2015–2020^a^

Type of disorder	Food security level,^b^ AOR (95% CI)		
	Marginal	Low	Very Low
**Metabolic**			
Obesity	1.33 (1.12–1.58)^c^	1.39 (1.17–1.65)^c^	1.42 (1.16–1.73)^c^
Diabetes	1.60 (1.17–2.18)^c^	1.54 (1.25–1.89)^c^	1.64 (1.23–2.19)^c^
Hyperglycemia	1.75 (1.36–2.25)^c^	1.45 (1.10–1.91)^c^	1.31 (0.97–1.77)
Hypertension	1.33 (1.12–1.59)^c^	1.26 (1.03–1.53)^c^	1.30 (1.07–1.58)^c^
**Renal**			
Weak or failing kidneys	1.70 (1.21–2.38)^c^	1.84 (1.17–2.88)^c^	2.26 (1.41–3.64)^c^
**Cardiovascular**			
Congestive heart failure	1.61 (1.14–2.29)^c^	2.15 (1.14–3.46)^c^	3.69 (2.11–6.44)^c^
Coronary heart disease	1.85 (1.22–2.81)^c^	1.68 (1.06–2.65)^c^	3.43 (1.86–6.33)^c^
Angina	1.63 (1.06–2.52)^c^	2.25 (1.46–3.47)^c^	1.53 (0.77–3.05)
Heart attack	1.89 (1.16–3.07)^c^	2.23 (1.49–3.32)^c^	3.21 (2.00–5.16)^c^
Stroke	1.33 (0.92–1.92)	2.29 (1.59–3.30)^c^	2.54 (1.53–4.22)^c^

Abbreviations: AOR, adjusted odds ratio; CKM, cardiovascular-kidney-metabolic; NHANES, National Health and Nutrition Examination Survey.

a Full food security was used as the reference group. All AORs were adjusted for age, sex, race and ethnicity, and socioeconomic status.

b Food security status was assessed by using the 18-item US Food Security Survey Module for households with children younger than 18 years and a shortened 10-item version for households without children. Participant responses were scored by NHANES staff and tallied to generate a raw food security score. A 4-level household food security variable was created, with 1 being full food security, 2 being marginal food security, 3 being low food security, and 4 being very low food security.

c Significant at *P* < .05.

Similar trends were observed for CVD, where participants with very low food security had the highest odds of congestive heart failure, (AOR = 3.69; 95% CI, 2.11–6.44; *P* < .001), coronary heart disease (AOR = 3.43; 95% CI, 1.86–6.33 *P* < .001), heart attack (AOR = 3.21; 95% CI, 2.00–5.16; *P* < .001), and stroke (AOR = 2.54; 95% CI, 1.53–4.22; *P* < .001) compared with those with full food security (Table 2).

When examining the cumulative association of CVDs with food insecurity, we found a stepwise increase in the probability of reported low or very low versus full or marginal food security with an increase in the number of CVDs (Figure **)**. The highest odds of low or very low versus full or marginal food security were observed among participants with 4 CVDs (AOR = 3.93; 95% CI, 1.91–8.08; *P* < .001), followed by those reporting 3 CVDs (AOR = 2.54; 95% CI, 1.41–4.56; *P* = .003).

**Figure:**
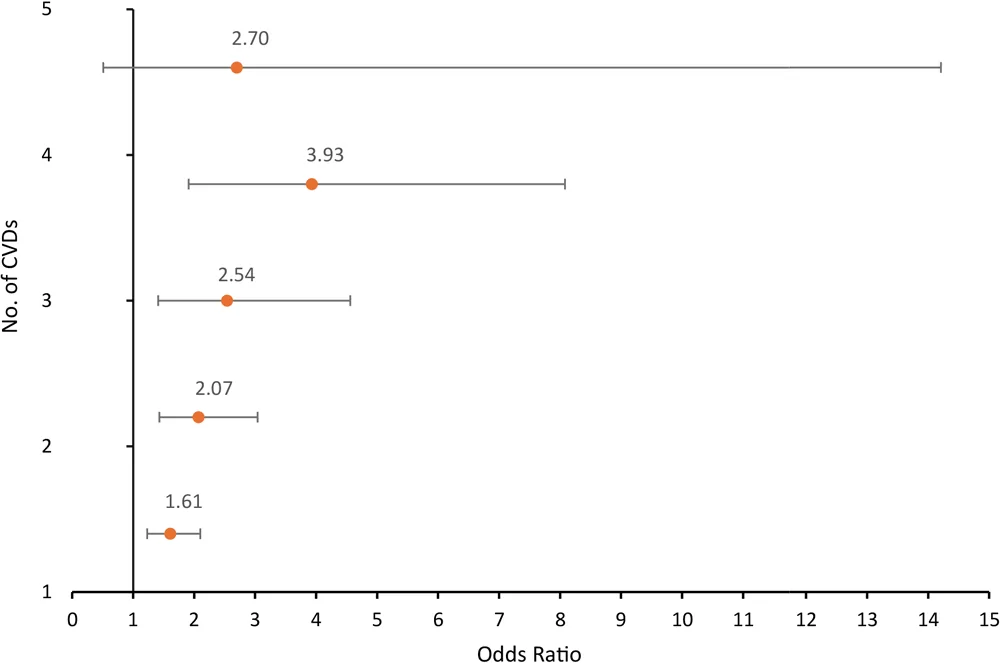
Cumulative association of CVDs with food insecurity, National Health and Nutrition Examination Survey, 2015–2020. Abbreviation: CVD, cardiovascular disease.

**Table:** 

No. of CVDs	Odds ratio (95% CI)
1	1.61 (1.23–2.1)
2	2.07 (1.43–3.04)
3	2.54 (1.41–4.56)
4	3.93 (1.91–8.08)
5	2.70 (0.51–14.21)

## Discussion

Consistent with prior US-based research, findings from this cross-sectional study consistently demonstrated significant, positive associations between food security and CKM syndrome–related conditions, especially obesity, diabetes, coronary heart disease, congestive heart failure, heart attack, and stroke. These associations persisted even after adjusting for demographic and SES factors, highlighting food security as an upstream correlate to incident or persisting chronic diseases (22).

This study noted a significantly higher prevalence of CVDs among those with low and very low food security, with a nearly 2- to 3-fold increase in the likelihood of CVDs among those with very low versus full food security. Furthermore, significant increases were seen in the likelihood of low and very low food security with an increase in the number of CVDs, thus confirming a bidirectional relationship between food security and CKM syndrome–related conditions. These findings are consistent with a previous NHANES analysis (1999–2018) that highlighted the continued prevalence of CVD among people with food insecurity (23) and the strong correlation between cumulative CVD prevalence and the compounding effect of reduced food security. Furthermore, participants with reduced food security were more likely to have metabolic and renal disorders, including diabetes, obesity, hypertension, and weak or failing kidneys. For renal disorders in particular, these findings align with prior research linking food insecurity with an increased likelihood of developing end-stage renal disease among adults with CKD, and CKD among participants with diabetes or hypertension (24,25).

The associations observed in this study likely reflect a constellation of interconnected biologic, behavioral, and structural mechanisms linking food insecurity with cardiometabolic, kidney, and CVD prevalence (6,26). At the structural level, features of the food environment, such as food deserts (an area with limited access to healthy food) and food swamps (an area with an abundance of unhealthy food options relative to healthier choices), limit access to affordable, nutritious food while promoting reliance on energy-dense, ultraprocessed foods that are high in sodium, refined carbohydrates, and unhealthy fats (25–27). Furthermore, the repeated cycle of food scarcity followed by overeating when food is available further disrupts metabolic processes, while the stress surrounding meal uncertainty provokes neuroendocrine and inflammatory responses (28). Current evidence suggests a positive relationship between such maladaptive behaviors and the development of cardiometabolic conditions like obesity, insulin resistance, hypertension, and dyslipidemia, which collectively contribute to the development of CKM syndrome (29). Additionally, food insecurity is closely linked to structural barriers such as limited health care access, medication nonadherence, and delayed disease detection, which may compound disease burden over time (30). Together, these elements provide a clear framework of how food insecurity is linked to the clustering of cardiometabolic, kidney, and CVDs observed in this study. Additionally, studies have shown that childhood food insecurity and socioeconomic disadvantage often persist into adulthood and thus remain substantial risk factors for several chronic diseases across the life course (31).

National trends further emphasize the urgency of addressing food insecurity. Although the US Department of Agriculture (USDA) reported a decline in food insecurity from 14.9% in 2011 to 10.5% in 2019, rates spiked during the COVID-19 pandemic, reaching 23% by April 2020 (26). Reports suggest that households with low income, low educational attainment, single mothers, changes in employment status, and lack of access to personal or public transportation have a higher prevalence of food insecurity. The USDA also reported that people with very low food security experience not only a higher prevalence of CVDs but also an elevated risk of death, reinforcing the broader public health implications of persistent and worsening food security (26). Promisingly, intervention studies have found that participation in produce prescription programs that provide free or discounted produce facilitates improvement in dietary quality and choices, leading to improved metabolic health outcomes (32). Collectively, the findings from this study build on prior evidence to highlight the critical role of food security in the development of chronic cardiometabolic and renal diseases among US adults and the potential public health benefits that may come from supporting and expanding current safety-net food assistance and produce prescription programs across the country.

This study has strengths and limitations. The use of a large, nationally representative dataset with rigorous data collection methods, including standardized biomarker assessments and in-person measurements, enhanced the reliability and generalizability of the study’s findings. Additionally, the use of weighted logistic regression to account for the complex survey design of NHANES improved statistical precision. However, the cross-sectional nature of the study limited causal inferences, as temporality between food insecurity and CKM syndrome–related outcomes could not be established. Reliance on self-reported data for certain health conditions (eg, CVD, weak or failing kidneys) may have introduced potential reporting bias. Despite these limitations, this study provides valuable insights into the associations between food insecurity and a novel, emerging collection of cardiometabolic and renal disorders.

Our study found a significant association between food insecurity and adverse cardiometabolic, renal, and cardiovascular health outcomes, emphasizing the critical role of socioeconomic factors related to patterns of disease risk. It underscores the urgent need for tailored public health interventions and policies to reduce food insecurity, given its observed association with chronic disease prevalence and health disparities. Addressing food insecurity through policy reforms, nutritional assistance programs, and health care integration strategies may support efforts to address long-term health disparities and provide insight relevant to the burden of CKM syndrome–related disorders in US populations.

## Appendix Distribution of Participant Characteristics, by NHANES Survey Year, Analysis of Association Between Cardiovascular-Kidney-Metabolic Syndrome and Food Insecurity, US, 2015–2020^a^

**Table:**

Characteristic	Overall (N = 13,937)	2015–2016 (N = 5,552)	2017–2020 (N = 8,385)
Age, mean (SE), y	47.4 (0.4)	47.3 (0.6)	47.5 (0.6)
BMI, mean (SE), kg/m^2^	29.6 (0.2)	29.4 (0.3)	29.7 (0.2)
**Sex**			
Male	6,726 (48.1)	2,664 (48.0)	4,062 (48.1)
Female	7,211 (51.9)	2,888 (52.0)	4,323 (51.9)
**Race and ethnicity**			
Hispanic	3,548 (15.8)	1,691 (15.3)	1,857 (16.1)
Non-Hispanic Asian	1,634 (5.7)	647 (5.7)	987 (5.7)
Non-Hispanic Black	3,400 (11.3)	1,197 (11.5)	2,203 (11.2)
Non-Hispanic White	4,734 (63.3)	1,805 (63.8)	2,929 (63.0)
Other/multiple races	621 (3.9)	212 (3.8)	409 (4.0)
**Family income-to-poverty ratio**			
At or below poverty line	2,758 (13.8)	1,192 (14.7)	1,566 (13.3)
Above poverty line	10,080 (86.2)	3,941 (85.3)	6,139 (86.7)
**Cardiometabolic disorders**			
Obesity	5,674 (40.9)	2,186 (39.6)	3,488 (41.7)
Type 2 diabetes	2,391 (13.5)	904 (12.8)	1,487 (13.9)
Hyperglycemia	3,881 (58.9)	1,468 (58.5)	2,413 (59.8)
Weak or failing kidneys	546 (3.2)	217 (3.4)	329 (3.1)
Hypertension	4,428 (29.9)	1,734 (30.1)	2,694 (29.7)
Congestive heart failure	498 (2.6)	192 (2.4)	306 (2.7)
Coronary heart disease	586 (3.9)	220 (3.4)	366 (4.3)
Angina	335 (2.3)	127 (2.1)	208 (2.4)
Heart attack	613 (3.6)	232 (3.3)	381 (3.8)
Stroke	612 (3.4)	194 (2.8)	418 (3.7)
**Food security^b^**			
Full	8,604 (71.7)	3,381 (71.2)	5,223 (71.9)
Marginal	2,051 (11.2)	800 (10.9)	1,251 (11.4)
Low	1,862 (9.5)	799 (10.1)	1,063 (9.1)
Very low	1,420 (7.7)	572 (7.9)	848 (7.6)

Abbreviations: NHANES, National Health and Nutrition Examination Survey.

^a^ Values are no. (weighted %), unless otherwise indicated. Not all categories add to values in column headers because not all survey respondents answered all questions.

^b^ Food security status was assessed by using the 18-item US Food Security Survey Module for households with children younger than 18 years and a shortened 10-item version for households without children. Participant responses were scored by NHANES staff and tallied to generate a raw food security score. A 4-level household food security variable was created, with 1 being full food security, 2 being marginal food security, 3 being low food security, and 4 being very low food security.

## References

[1] Lozano R , Naghavi M , Foreman K , Lim S , Shibuya K , Aboyans V , . Global and regional mortality from 235 causes of death for 20 age groups in 1990 and 2010: a systematic analysis for the Global Burden of Disease Study 2010. *Lancet.* 2012;380(9859):2095–2128. 10.1016/S0140-6736(12)61728-0 23245604 PMC10790329

[2] Ndumele CE , Neeland IJ , Tuttle KR , Chow SL , Mathew RO , Khan SS , ; American Heart Association. A synopsis of the evidence for the science and clinical management of cardiovascular-kidney-metabolic (CKM) syndrome: a scientific statement from the American Heart Association. *Circulation.* 2023;148(20):1636–1664. 10.1161/CIR.0000000000001186 37807920

[3] Ndumele CE , Rangaswami J , Chow SL , Neeland IJ , Tuttle KR , Khan SS , ; American Heart Association. Cardiovascular-kidney-metabolic health: a presidential advisory from the American Heart Association. *Circulation.* 2023;148(20):1606–1635. 10.1161/CIR.0000000000001184 37807924

[4] Reeder NK , Reneker JC . Food insecurity is associated with metabolic syndrome among US adults: NHANES 2005–2016. *Nutr Res.* 2024;126:159–166. 10.1016/j.nutres.2024.03.014 38718433 PMC11179963

[5] Liu J , Yi SS , Russo RG , Horowitz CR , Zhang D , Rajbhandari-Thapa J , . Trends and disparities in prevalence of cardiometabolic diseases by food security status in the United States. *Nutr J.* 2024;23(1):4. 10.1186/s12937-023-00910-4 38172928 PMC10763098

[6] Te Vazquez J , Feng SN , Orr CJ , Berkowitz SA . Food insecurity and cardiometabolic conditions: a review of recent research. *Curr Nutr Rep.* 2021;10(4):243–254. 10.1007/s13668-021-00364-2 34152581 PMC8216092

[7] American Heart Association. *AHA Clinical Slide Series. Adapted From: Cardiovascular-Kidney-Metabolic Health: A 2023 Presidential Advisory From the American Heart Association.* 2023. Accessed June 23, 2026. https://professional.heart.org/-/media/PHD-Files-2/Science-News/Clinical-Update-Slides/CKMH_Presidential_Advisory_slide_set_final.pdf

[8] Centers for Disease Control and Prevention. National Health and Nutrition Examination Survey 2017–March 2020 Data Documentation, Codebook, and Frequencies, Demographic Variables and Sample Weights (P_DEMO) 2025. Accessed June 23, 2026. https://wwwn.cdc.gov/Nchs/Data/Nhanes/Public/2017/DataFiles/P_DEMO.htm

[9] Centers for Disease Control and Prevention. CDC extended BMI-for-age growth charts. 2024. Accessed June 23, 2026. https://www.cdc.gov/growthcharts/extended-bmi.htm

[10] Centers for Disease Control and Prevention. NHANES survey methods and analytic guidelines. Accessed July 19, 2024. https://wwwn.cdc.gov/nchs/nhanes/analyticguidelines.aspx#sample-design

[11] Flack JM , Adekola B . Blood pressure and the new ACC/AHA hypertension guidelines. *Trends Cardiovasc Med.* 2020;30(3):160–164. 10.1016/j.tcm.2019.05.003 31521481

[12] Cleaveland Clinic. Hyperglycemia (high blood sugar). 2023. Accessed July 28, 2025. https://my.clevelandclinic.org/health/diseases/9815-hyperglycemia-high-blood-sugar

[13] Centers for Disease Control and Prevention. *National Health and Nutrition Examination Survey (NHANES): MEC Laboratory Procedures Manual.* 2017. Accessed July 14, 2026. https://wwwn.cdc.gov/nchs/data/nhanes/public/2017/manuals/2017_MEC_Laboratory_Procedures_Manual.pdf

[14] National Center for Health Statistics. National Health and Nutrition Examination Survey: 2017–2018 data documentation, codebook, and frequencies — diabetes (DIQ_J). 2020. Accessed April 2, 2025. https://wwwn.cdc.gov/Nchs/Data/Nhanes/Public/2017/DataFiles/DIQ_J.htm#DIQ010

[15] National Center for Health Statistics. National Health and Nutrition Examination Survey: 2017–2018 data documentation, codebook, and frequencies — kidney conditions — urology (KIQ_U_J). Accessed April 2, 2025. https://wwwn.cdc.gov/Nchs/Data/Nhanes/Public/2017/DataFiles/P_KIQ_U.htm#KIQ022

[16] National Center for Health Statistics. National Health and Nutrition Examination Survey: 2015–2016 data documentation, codebook, and frequencies — medical conditions (MCQ_I). 2017. Accessed April 2, 2025. https://wwwn.cdc.gov/Nchs/Data/Nhanes/Public/2015/DataFiles/MCQ_I.htm#MCQ160b

[17] US Department of Agriculture. Food security in the U.S. — survey tools. U.S. adult food security survey module. Accessed September 13, 2024. https://www.ers.usda.gov/topics/food-nutrition-assistance/food-security-in-the-u-s/survey-tools/#household

[18] US Department of Agriculture. Guide to measuring household food security (revised 2000). Office of Analysis, Nutrition, and Evaluation; 2000. Accessed June 23, 2026. https://www.fna.usda.gov/research/guide-measuring-household-food-security-revised-2000

[19] US Department of Health and Human Services, Office of the Assistan Secretary for Planning and Evaluation. 2021 Poverty guidelines. Accessed January 25, 2026. https://aspe.hhs.gov/2021-poverty-guidelines#threshholds

[20] Colin Bell A , Adair LS , Popkin BM . Ethnic differences in the association between body mass index and hypertension. *Am J Epidemiol.* 2002;155(4):346–353. 10.1093/aje/155.4.346 11836199

[21] Buford TW . Hypertension and aging. *Ageing Res Rev.* 2016;26:96–111. 10.1016/j.arr.2016.01.007 26835847 PMC4768730

[22] Sun Y , Liu B , Rong S , Du Y , Xu G , Snetselaar LG , . Food insecurity is associated with cardiovascular and all‐cause mortality among adults in the United States. *J Am Heart Assoc.* 2020;9(19):e014629. 10.1161/JAHA.119.014629 32975173 PMC7792411

[23] Brandt EJ , Chang T , Leung C , Ayanian JZ , Nallamothu BK . Food insecurity among individuals with cardiovascular disease and cardiometabolic risk factors across race and ethnicity in 1999–2018. *JAMA Cardiol.* 2022;7(12):1218–1226. 10.1001/jamacardio.2022.3729 36170056 PMC9520443

[24] Banerjee T , Crews DC , Wesson DE , Dharmarajan S , Saran R , Ríos Burrows N , ; CDC CKD Surveillance Team. Food insecurity, CKD, and subsequent ESRD in US adults. *Am J Kidney Dis.* 2017;70(1):38–47. 10.1053/j.ajkd.2016.10.035 28215947 PMC5765854

[25] Crews DC , Kuczmarski MF , Grubbs V , Hedgeman E , Shahinian VB , Evans MK , ; Centers for Disease Control and Prevention Chronic Kidney Disease Surveillance Team. Effect of food insecurity on chronic kidney disease in lower-income Americans. *Am J Nephrol.* 2014;39(1):27–35. 10.1159/000357595 24434743 PMC3952065

[26] Chang R , Javed Z , Taha M , Yahya T , Valero-Elizondo J , Brandt EJ , . Food insecurity and cardiovascular disease: current trends and future directions. *Am J Prev Cardiol.* 2022;9:100303. 10.1016/j.ajpc.2021.100303 34988538 PMC8702994

[27] Richardson AS , Boone-Heinonen J , Popkin BM , Gordon-Larsen P . Are neighbourhood food resources distributed inequitably by income and race in the USA? Epidemiological findings across the urban spectrum. *BMJ Open.* 2012;2(2):e000698. 10.1136/bmjopen-2011-000698 22505308 PMC3329604

[28] Towers AE , Freund GG . Nutritional psychoneuroimmunology: is the inflammasome a critical convergence point for stress and nutritional dysregulation? *Curr Opin Behav Sci.* 2019;28:20–24. 10.1016/j.cobeha.2019.01.014 31667204 PMC6820983

[29] Anand SS , Hawkes C , de Souza RJ , Mente A , Dehghan M , Nugent R , . Food consumption and its impact on cardiovascular disease: importance of solutions focused on the globalized food system: a report from the workshop convened by the World Heart Federation. *J Am Coll Cardiol.* 2015;66(14):1590–1614. 10.1016/j.jacc.2015.07.050 26429085 PMC4597475

[30] Seligman HK , Laraia BA , Kushel MB . Food insecurity is associated with chronic disease among low-income NHANES participants. *J Nutr.* 2010;140(2):304–310. 10.3945/jn.109.112573 20032485 PMC2806885

[31] Mobley C , Luo Y , Fernandez M , Hossfeld L . Social determinants of health and college food insecurity. *Nutrients.* 2024;16(9):1391. 10.3390/nu16091391 38732637 PMC11085391

[32] Hager K , Du M , Li Z , Mozaffarian D , Chui K , Shi P , . Impact of produce prescriptions on diet, food security, and cardiometabolic health outcomes: a multisite evaluation of 9 produce prescription programs in the United States. *Circ Cardiovasc Qual Outcomes.* 2023;16(9):e009520. 10.1161/CIRCOUTCOMES.122.009520 37641928 PMC10529680

